# Risk Assessment

**DOI:** 10.1007/s40140-018-0246-9

**Published:** 2018-01-30

**Authors:** Pragya Ajitsaria, Sabry Z. Eissa, Ross K. Kerridge

**Affiliations:** 1 Department of Anaesthesia & Perioperative Medicine, John Hunter Hospital, Locked Bag 1 HRMC, Newcastle, NSW 2310 Australia; 2 University of Newcastle, Newcastle, NSW Australia

**Keywords:** Preoperative evaluation, Preoperative risk assessment, Prehabilitation, Surgical risk assessment

## Abstract

**Purpose of Review:**

The central question of preoperative assessment is not “What can be done?” but “What should be done and how?” Predicting a patient’s risk of unwanted outcomes is vital to answering this question. This review discusses risk prediction tools currently available and anticipates future developments.

**Recent Findings:**

Simple, parsimonious risk scales and scores are being replaced by complex risk prediction models as high-capacity information systems become ubiquitous. The accuracy of risk estimation will be further increased by improved assessment of physical fitness, frailty, and incorporation of existing and novel biomarkers. However, the limitations of risk prediction for individual patient care must be recognized.

**Summary:**

Risk prediction is transforming from clinical estimation to statistical science. Predictions should be used within the context of a patient’s baseline risk (life expectancy independent of surgery), personal circumstances, quality of life, their expectations and values, and consideration of outcomes that are meaningful for the patient.

## Introduction

Perioperative risk assessment and outcome prediction are growing in importance and scope of application throughout the perioperative process, from contemplation of surgery through to postoperative recovery and rehabilitation.

Risk estimation is used to guide appropriate investigations, to facilitate the identification and quantification of potential to improve preoperative patient parameters, and to help plan preoperative preparation [1, 2]. High-risk patients may be diverted from inappropriate surgical interventions. Alternatively, the beneficial effects of preoperative interventions such as optimization of medical therapy, fitness training, weight loss, or smoking cessation can be individualized for more personalized patient care.

Standardizing risk estimation can aid preoperative discussion and planning among treating teams by creating a common language of risk understood by surgical, anesthetic, intensive care, and internal medicine teams alike. Preoperative risk estimation can also provide the basis for predicting service demand, planning services such as ICU bed requirements, risk-adjusted benchmarking, and quality improvement of patient care systems.

More accurate and meaningful estimates of patient-centered risk will enable better shared decision-making between clinicians and patients regarding treatment options. Communicating risk in terms of natural frequencies rather than statistical risk descriptions commonly used in medical literature can assist with comprehensive discussions with patients regarding risks and benefits of proceeding with surgery or choosing non-operative pathways.

When considering perioperative risk, we must also be cognizant of which outcomes are being considered. Assessment of risk in terms of mortality and length of stay is no longer sufficient to describe postoperative outcomes, particularly for the older and frailer population of today [3, 4]. To this end, much work is currently being done to establish and standardize perioperative outcomes that are meaningful to surgical patients [5••].

## Risk Stratification Tools

Scoring systems have an established role in producing a numerical probability of specified outcomes for a patient. However, an accurate numerical probability of particular outcomes for an “average” patient has limited usefulness in the individual patient setting. The presentation of risk as a mathematical score may give a reassuring, but unfounded, appearance of certainty. The particular circumstances of a planned operation and factors specific to the individual patient, some of which may be subtle or even unrecognized, must be considered.

Various tools are available to enable the estimation of perioperative risk for both planned and emergency surgeries [6, 7]. They may be Categorical risk scales, Risk scores, or Risk prediction models. They may be generic (i.e., for any type of surgery) or may be specific for particular types of surgery (e.g., cardiac, colorectal, neurosurgical, or hepatic). They may focus on global adverse outcomes (death, prolonged hospitalization) or on particular complications (e.g., postoperative major adverse cardiac events [MACE], respiratory failure, acute kidney injury (AKI), or postoperative nausea and vomiting).

Categorical risk scales, such as the ASA PS scale, are simple to use and may be robust enough to describe the patient’s overall risk for purposes such as for clinical communication or audit. They have limited application for predicting outcomes in an individual patient [8].

Risk scores incorporate a limited number of independent predictors of outcomes, scored and weighted. Risk scores can be surgery specific, and thus most appropriate for high-risk surgeries, where surgical factors are the major determinants of patient outcomes (e.g., cardiac, hepatic, or esophageal surgery). More commonly, they are outcome based, where patient factors are more important influences on outcome than specific surgical factors. Unsurprisingly, the development of scoring systems to estimate a patient’s cardiac risk for non-cardiac surgery has been the focus of greatest attention [9]. The well-known Lee Revised Cardiac Risk Index (RCRI) is an example [10]. Other disease-specific risk scores have been developed for predicting respiratory failure, renal injury, or other specific complications postoperatively [11]. A number of other published preoperative risk prediction scores, such as SORT and the Surgical Apgar Score, have been designed with parsimonious data requirements in order to be simple and easy to use [12, 13].

Risk scoring systems may have reasonable predictive value, but are constrained by their own simplicity, by treatment changes over time, and by population or institutional differences. Accuracy for use with individual patients is limited [14].

Risk prediction models use a large dataset composed of data from multiple individual patients to predict the probability of a variety of outcomes for an individual patient. Recent and ongoing advances in informatics (“big data”) enable routine real-time incorporation of multiple variables into risk prediction models and make the use of these models in routine clinical practice feasible. Nevertheless, the scientific limitations to the use of these models when applied to individual patients still apply, and clinical caution remains appropriate [6, 7].

## Factors Predicting Patient Outcome

The major predictors of patient outcome are patient age, functional status (physical fitness), comorbidities such as cardiorespiratory disease and diabetes, abnormalities of biomarkers such as hemoglobin (Hb), albumin and renal function, and inflammatory markers. Recently, the importance of considering patient frailty as a factor predicting outcomes in the perioperative setting has been recognized. Unplanned/acute surgery has consistently been found to have three or more times or more risk than planned surgery [15, 16•].

## Measures of Functional Status (i.e., Physical Fitness)

The importance of functional status as a predictor of survival after surgery has been apparent for many years [17]. However, the patient and clinician’s subjective impressions of functional status or metabolic equivalents (METs) are often inaccurate [18•, 19]. Even with semi-objective measures such as stair climbing, direct observation of physical activity is important and can provide a stronger basis for discussion with the patient as part of shared decision-making before surgery. Cardiopulmonary exercise testing (CPET) is discussed in the following section.

## Frailty Assessment

Frailty has traditionally been considered to represent decreased or limited capacity to maintain homeostasis at times of physiological insult. The world is aging, such that by 2050, the population over 80 years old will increase threefold [20]. Given the association between age and frailty, perioperative frailty assessment has recently become a growing focus of interest. Validated frailty scoring systems can now be used in the older preoperative surgical candidate to predict meaningful morbidity and mortality [21].

Multiple frailty scoring systems exist, broadly categorized into the “Frailty Phenotype” and the “Deficit Accumulation Model.” Frailty Phenotype is based on assessment of unintentional weight loss, grip strength, self-reported exhaustion, gait speed, and low physical activity [22, 23]. The Deficit Accumulation Model summates the number of deficits an individual has accumulated across a number of domains including illness and activities of daily living [24]. Studies demonstrating the correlation of scores with increased morbidity and mortality are growing, but it remains unclear which score is the “best” to use in the clinical setting. It is also yet to be established if frailty is modifiable or optimizable.

Current options for assessing frailty include the original frailty scores (phenotype or deficit accumulation models), mixed models (such as combining Katz score, Charlson Index, timed get up and go, albumin, anemia, Mini-Cog score, and recent fall), independent surrogate markers (such as gait speed and grip strength), or more complex modeling based on matching frailty markers against the preoperative variables in the National Surgical Quality Improvement Program (NSQIP) database to identify “simplified” frailty indices [22–28].

The “cachexia” and “sarcopenia” syndromes are considered to overlap the frailty syndrome in the elderly [21]. Independent measures of muscle mass such as serum C1q and computerized tomography (CT) imaging for cross-sectional area of psoas muscle are also gaining attention as possible predictive markers of frailty and complications [29, 30].

## Cardiopulmonary Exercise Testing

CPET has recently gained attention as a possible objective measure of a patient’s exercise capacity [31]. In the UK, preoperative CPET assessment is now widely used in a majority of surgical centers, particularly to guide allocation of postoperative intensive care resources after major surgery [32, 33].

CPET results can be used to inform decision-making such as whether to proceed with major surgery, or to choose a less aggressive alternative. Traditionally, the maximum rate of oxygen consumption (VO_2_) max has been the parameter found to be the most useful in guiding clinical decision-making for patients deemed borderline for lung resection surgery, with a VO_2_ of less than 15 ml/kg/min deemed a high risk patient [34]. Anaerobic threshold (AT) is another parameter measured by CPET, indicating the point in exercise where a systemic lactic acidosis occurs. Since the original work by Older and colleagues, a number of other small studies have confirmed that a low anaerobic threshold (< 9–11 ml/kg/min) preoperatively is associated with worse outcomes following major surgery, including morbidity, mortality, and length of stay [35–37]. Other variables such as the ventilatory equivalents for CO_2_ (VE/VCO_2_), which measure ventilatory efficiency, also show promise in predicting risk both independently or in combination with AT and VO_2_ max [38, 39•]. Despite popularity in the UK, the evidence for CPET use as a predictor of adverse outcomes is inconclusive. Large multicenter trials currently in progress may help clarify this issue [40].

Evolving roles for CPET include guiding preoperative optimization and timing of surgery, particularly in patients with cancer being treated with neoadjuvant therapy. Deterioration of a patient’s physical fitness during preoperative chemo or radiation therapy is well described, may be partly reversible, and is multifactorial [41, 42•, 43••]. Using CPET to understand the patients’ pathophysiology and to guide directed optimization (e.g., iron infusions or prehabilitation) is novel and may improve outcomes in certain patient groups [43••]. Hybrid preoperative scoring systems using CPET data are also showing promise in predicting risk and for planning postoperative care [44••].

## Cardiac Assessment

For many years, assessment of a patient’s cardiac risk has focused on ischemic heart disease, partly based on the presumption that the risk of perioperative MACE can be reduced by preoperative interventions including revascularization and/or pharmacotherapy. The American College of Cardiology/American Heart Association (ACC/AHA) guidelines (and the similar European Society of Cardiology [ESC] guidelines) provide a framework for preoperative testing and optimization of a patient’s cardiac status [45, 46]. The similarity of these guidelines represents a reasonably stable consensus on assessment with regard to ischemic heart disease. The use of a validated tool to predict the risk of perioperative MACE is recommended for the assessment of patients undergoing non-cardiac surgery, but a more complex assessment and intervention is only recommended if it is likely to change patient management. The RCRI and the NSQIP-derived Gupta score are the most validated risk prediction tools, although modifications are in progress [47]. The recent Canadian Cardiovascular Society (CCS) Guidelines emphasizing the use of biomarkers present an alternative approach (discussed in the following section) [48•].

## Biomarkers for Perioperative Risk Assessment

Given the ubiquity of blood testing preoperatively, it is unsurprising that the incorporation of objective measures characterizing derangement in patient physiology is a growing focus in risk assessment. A number of biomarkers have been studied, all showing promise in improving the accuracy of risk prediction.

### Albumin

Measured preoperative serum albumin < 30 g/l has been known for a number of years to be associated with increased perioperative mortality risk [15]. A large recent study confirms the importance of serum albumin for predicting complications following surgery, even at levels deemed to be only moderately low (30–35 g/l) [49•]. Postoperative albumin reductions also appear to increase risk of complications such as AKI, irrespective of higher preoperative levels [50, 51]. This may reflect individual, deleterious responses to a surgical insult and highlight the importance of perioperative albumin measurement [50].

### Hemoglobin

Anemia is strongly predictive of adverse outcomes following surgery [52]. Preoperative correction of anemia is a well-established method of optimization. Cardiovascular performance can be improved by the administration of allogenic red blood cells, although this strategy would not be recommended given its implications for perioperative risk [53, 54]. Recent advances in intravenous iron therapy provide a preferable means of correcting both iron deficiency and anemia [55]. A recent study suggests that total Hb mass, as opposed to Hb concentration alone, appears to be the most influential to cardiovascular performance measured by CPET [56•]. This suggests that expansion of blood volume and overall Hb mass could be a therapeutic target in the future [56•].

### HbA1c and Diabetes

The risks associated with surgery are elevated in patients with diabetes mellitus. Both the prevalence of diabetes and the risk of complications are increased in patients having cardiac, vascular, or major orthopedic surgery [57]. Furthermore, the population prevalence of diabetes is increasing worldwide. There are limited high-quality studies supporting routine preoperative testing using blood glucose or HbA1c (glycated hemoglobin) in otherwise healthy adult patients undergoing elective non-cardiac surgery [58]. However, using HbA1c as a screening tool is justified prior to major orthopedic and vascular surgery and should be considered in other patients [59].

### Troponin, Natriuretic Peptides, and Copeptin in Cardiac Risk Assessment

The appropriate role of cardiac biomarkers such as troponin and natriuretic peptides such as beta natriuretic peptide (BNP) and N-terminal-BNP(NT-BNP) as predictors of patient risk is controversial [60, 61]. In a population, there is an association between raised (resting) troponin and cardiac disease [62]; preoperative raised troponin may be used as a predictor of postoperative adverse outcomes [63]. However, troponin may be raised by heavy cardiac workload without evidence of long-term cardiac injury [64]. In major surgical patients, raised postoperative troponin is predictive of poorer long-term outcomes [65].

The use of preoperative BNP and postoperative troponin has been strongly endorsed in the 2017 CCS Guidelines on Perioperative Cardiac Risk Assessment [48•]. However, the perioperative use of BNP needs more widespread validation. The CCS guideline represents a markedly different approach to that advocated by the current guidelines from the equivalent authoritative groups in the USA and Europe: The US-based guidelines do not incorporate BNP testing and currently recommend against routine postoperative troponin assay. The perioperative interventions described in the current CCS guidelines are limited to preoperative risk discussion for patient decision-making, and changes to postoperative management such as shared care postoperatively, or long-term secondary cardiac risk prevention therapies. The use of biomarkers to guide preoperative interventions such as prehabilitation, or to modify intraoperative care, is not considered. There is no incorporation of intraoperative observations or events to modify risk assessment.

Copeptin is a peptide cleaved from vasopressin precursor peptide and is thus a marker of endogenous stress [66]. It has shown value in diagnosis and prognosis of myocardial injury in the non-surgical patient. It has recently been evaluated as a biomarker in the perioperative setting and may have incremental value to improve preoperative risk stratification and prediction of myocardial injury in non-cardiac surgery [67•]. Renal interaction may reduce accuracy of diagnosis [68].

Although promising, the appropriate role and perioperative use of troponin, BNP, copeptin, or other novel biomarkers to substantially improve risk prediction or modify therapeutic interventions requires further evaluation [69].

### Biomarkers for Renal Risk Assessment

Traditionally, preoperative creatinine concentration has been used to predict perioperative AKI, with a recent large prospective study confirming its value in risk modeling[70]. Creatinine responds slowly to acute changes in kidney function, and the search for novel biomarkers has been in earnest for a number of years [71, 72]. Clinical utility of several biomarkers is now a reality in the perioperative setting [73, 74]. Some of these show promise for more rapid diagnosis of AKI and include neutrophil gelatinase-associated lipocalin (NGAL), cystatin C, liver fatty acid-binding protein (L-FABP), and interleukin 18 [73, 74].

## Considerations for Specific Risk Systems

### P-POSSUM

The P-POSSUM Scoring system is a surgical audit tool that uses 12 physiological and 6 operative variables to compare outcomes in surgical patients [75]. While the system has been externally validated in multiple settings, its use to predict risk for individual patients is cautioned against [76]. It has limited applicability for early preoperative risk prediction, since the most critical data points apply to operative findings. It may be useful for guiding decision-making postoperatively with regard to admission to an intensive care unit (ICU), or as an indicator for increased postoperative surveillance.

### The ACS NSQIP Risk Prediction Model

The American College of Surgeons National Surgical Quality Improvement Program (ACS NSQIP) has recently developed a generic Surgical Risk Calculator (SRC). The SRC compares 21 preoperative patient variables to a large NSQIP database of patient outcomes to predict the risk of postoperative complications [77]. A useful facility within the program is the capacity to share the predictions in a “patient-friendly” format that facilitates discussion for shared decision-making purposes. Following an update in 2016, the database contains data from over 2.7 million patients and 600 hospitals. The SRC is freely available online (http://riskcalculator.facs.org).

The validation of the ACS SRC has shown good accuracy at predicting outcomes such as MACE and pneumonia [78, 79]. The tool has not been validated for use outside of the USA. Other limitations include the substantial effect ASA-PS grade, which is included in the calculator, has on calculated risk, with the potential for inter-rater variability. The database also does not incorporate findings at the time of surgery and is not dynamic to incorporate change in risk predictions after the development of postoperative complications.

The SRC is continuing to develop and it is hoped that some of the disadvantages mentioned will be addressed in future versions. Recent hybrid modeling with incorporation of biomarkers and surgery-specific factors already appears to have improved its accuracy [80, 81]. It is difficult to envisage another risk prediction modeling system being developed that would have the existing advantages of the ACS SRC. With current advances in information systems, there will be little reason to use less complex risk scores.

## Future Developments: Risk Stratification in the Era of “Big Data”

Until recently, clinical risk prediction tools have been limited by the workload required to collect and process clinical data. This has limited both the size and timeliness of clinical datasets. Clinical data can now be continuously collected and processed and analyzed by modern informatics systems. In the near future, data collection and aggregation will become ubiquitous. Data linkage and data mining will produce new sources of information for research, including identification of previously unrecognized patterns of clinical presentations.

Accurate gathering of patient data preoperatively will be the foundation of perioperative risk assessment in big data systems. This will drive a requirement for systematic, accurate recording of a wide number of data points including disease comorbidities, smoking, obesity, fitness, social factors, family history, and, ultimately, genetic data.

Risk prediction models based on sophisticated “artificial intelligence” systems may incorporate “real-time” intra- and postoperative physiological and laboratory data, with dynamic and ongoing modification of risk prediction as a result. This may include prediction of outcomes such as death, unplanned ICU admission, or the need for Rapid Response Team intervention [82, 83]. Risk predictions may form the basis of automated warnings to staff [84].

## Conclusion

In the future of patient-centered health care, the risks of proceeding with surgery must be quantified using definitions that are transparent and meaningful to all involved stakeholders [85]. However the risks of not proceeding with surgery must also be considered.

Within this context, the capacity of clinical information systems to upload, integrate, and analyze massive amounts of data from disparate sources is developing rapidly. It must be anticipated that risk prediction models and the prediction of long-term survival and quality of life will evolve exponentially. This will dramatically change clinical practice in the near future.
